# NUDIX hydrolases with inorganic polyphosphate exo- and endopolyphosphatase activities in the glycosome, cytosol and nucleus of *Trypanosoma brucei*

**DOI:** 10.1042/BSR20190894

**Published:** 2019-05-17

**Authors:** Ciro D. Cordeiro, Michael A. Ahmed, Brian Windle, Roberto Docampo

**Affiliations:** 1 Center for Tropical and Emerging Global Diseases, University of Georgia, Athens, GA 30602, USA; 2 Department of Cellular Biology, University of Georgia, Athens, GA 30602, U.S.A.

**Keywords:** glycosome, inorganic polyphosphates, nucleus, polyphosphatase, trypanosomes

## Abstract

*Trypanosoma brucei*, a protist parasite that causes African trypanosomiasis or sleeping sickness, relies mainly on glycolysis for ATP production when in its mammalian host. Glycolysis occurs within a peroxisome-like organelle named the glycosome. Previous work from our laboratory reported the presence of significant amounts of inorganic polyphosphate (polyP), a polymer of three to hundreds of orthophosphate units, in the glycosomes and nucleoli of *T. brucei*. In this work, we identified and characterized the activity of two Nudix hydrolases (NHs), *T. brucei* Nudix hydrolase (TbNH) 2 and TbNH4, one located in the glycosomes and the other in the cytosol and nucleus, respectively, which can degrade polyP. We found that TbNH2 is an exopolyphosphatase with higher activity on short chain polyP, while TbNH4 is an endo- and exopolyphosphatase that has similar activity on polyP of various chain sizes. Both enzymes have higher activity at around pH 8.0. We also found that only TbNH2 can dephosphorylate ATP and ADP but with lower affinity than for polyP. Our results suggest that NHs can participate in polyP homeostasis and therefore may help control polyP levels in glycosomes, cytosol and nuclei of *T. brucei*.

## Introduction

Inorganic polyphosphate (polyP) is a linear polymer of phosphate (orthophosphate [P_i_]) that can range from three to hundreds of P_i_ units. PolyP has been found in most species investigated, from bacteria to animals [1]. In *Trypanosoma brucei*, one of the agents of African trypanosomiasis, polyP is synthesized by the vacuolar transporter chaperone (VTC) complex [2], which is located in the acidocalcisome [3], an acidic organelle that stores calcium and other cations together with P_i_, inorganic pyrophosphate (PP_i_), and polyP [4]. Hydrolysis of polyP with release of P_i_ occurs by the activity of a cytosolic exopolyphosphatase (PPX) [5] and by the PPX activity of the acidocalcisome vacuolar soluble pyrophosphatase (VSP) [6–9]. No endopolyphosphatase (PPN) activity, which cleaves internal phosphoanhydride bonds generating shorter polyP molecules, like the yeast PPN1 (YDR452W) [10,11] and PPN2 (YNL217W) [12], has yet been reported in trypanosomatids.

A third endopolyphosphatase that has been described in yeast is diadenosine and diphosphoinositol polyphosphate phosphohydrolase (Ddp1) (YOR163W) [13], initially described as diadenosine hexaphosphate and diphosphoinositol polyphosphate hydrolase (DIPP) [14,15]. Ddp1 is localized in the cytosol and nucleus [16] and belongs to the Nudix (nucleoside diphosphate-linked moiety X) hydrolase family, which is characterized by a MuT motif or Nudix box of 23 amino acids (GX_5_EX_7_REUXEEXGU) where U is a bulky aliphatic residue and X is any amino acid [17]. The glutamic acid residues within the Nudix box binds to divalent cations cofactors like Mg^2+^ and Mn^2+^ [18]. Ddp1 and its human homologs DIPPs, DIPP1, DIPP2 and DIPP3, have polyP endopolyphosphatase activity [13]. Interestingly, the yeast and human enzymes also have 5-diphosphoinositol pentakisphosphate (5-IP_7_) hydrolase activity that helps to regulate inositol pyrophosphate signaling [13].

The Nudix superfamily (Pfam PF00293) is found in archaea, bacteria, eukaryotes and viruses and includes pyrophosphohydrolases of nucleotide sugars and alcohols, nucleoside and deoxynucleoside triphosphates ([d]NTPs), dinucleoside polyphosphates, dinucleotide coenzymes and capped RNAs [19]. *Trypanosoma brucei* has five Nudix proteins, of which two have been characterized. Nudix Hydrolase 1 (*T. brucei* Nudix hydrolase [TbNH] 1 or MERS1) binds to the RNA–editing complex and helps stabilizing edited mRNAs [20]. Nudix Hydrolase 4 (TbNH4 or TbDcp2) is a mRNA de-capping enzyme that removes the 5′ cap from processed mRNAs [21].

In this work, we investigated the ability of *T. brucei* Nudix hydrolases (NHs) to hydrolyze polyP and 5-IP_7_. We identified two polyphosphatases, TbNH2 and TbNH4, with polyP exopolyphosphatase and endopolyphosphatase activities, respectively. TbNH4 is the first trypanosome endopolyphosphatase class of enzyme described. TbNH2 localizes to the glycosomes while TbNH4 localizes to the cytosol and nucleus, results which are consistent with our recent demonstration of polyP in the glycosomes and nucleoli of these parasites [22]. None of the enzymes hydrolyzes 5-IP_7_.

## Materials and methods

### Materials

Chemically synthesized 5-diphosphoinositol pentakisphosphate [23] was provided by Dr. Henning Jessen, Albert-Ludwigs-University of Freiburg, Germany. PolyP_60_ was a gift from Dr. Toshikazu Shiba (RegeneTiss Inc., Tokyo, Japan). PolyP_700_ was purchased from Kerafast Inc. (Boston, MA, U.S.A.). The plasmid for expression of human DIPP was a gift from Dr. Dorothea Fiedler (Humboldt University of Berlin, Germany). Monoclonal antibody against phosphate pyruvate dikinase (PPDK) was a gift from Dr. Frédéric Bringaud (University of Bordeaux, France).

### Cell cultures

*Trypanosoma brucei* procyclic form (PCF) Lister 427, 29-13 TetR/T7RNAP cell line was used. Procyclic cells were cultivated at 28°C in SDM-79 [24] supplemented with 10% heat-inactivated FBS and hemin (7.5 µg/ml). Drug concentrations used for selection and maintenance of procyclic cell lines were: hygromycin (50 µg/ml), G418 (15 µg/ml), and blasticidin S (5 µg/ml).

## Methods

### Cloning, primers, expression and SDS/PAGE

The sequences of TbNH1 (Tb927.11.15640), TbNH2 (Tb927.5.4350), TbNH3 (Tb927.11.9810), TbNH4 (Tb927.6.2670) and TbNH5 (Tb927.10.4680) were amplified from genomic DNA by PCR (Supplementary Table S1) and cloned in expression vector pET32 Ek/LIC (Novagen) following manufacturer instructions. Constructs inserts were verified by Sanger sequencing and then transformed in *Escherichia coli* BL21-CodonPlus (DE3). Protein expression was induced by addition of 0.1 mM isopropyl β-D-1-thiogalactopyranoside (IPTG) to bacterial cultures in Luria Bertani broth shaking for 2 h at 25°C. Culture was chilled on ice and bacteria harvested by centrifugation. Pellet was suspended in 20 mM Tris HCl, 150 mM NaCl, pH 7.4 with protease inhibitors (Sigma P8465) and sonicated on ice. Lysate was then centrifuged 15000 × ***g*** for 30 min and then filtered on 0.8 µm syringe filter units (Millipore). Protein purification was performed using Nickel column HIS-Select^®^ Cartridges as recommended by manufacturer. Elution fractions with purified protein were dialyzed, replacing elution buffer for 300 mM NaCl, 200 mM Tris HCl, pH 7.4 with 20% glycerol. Expression was verified by SDS/PAGE followed by Coomassie blue staining and protein aliquots were stored at −80°C until further use. Protein concentration was determined using Pierce BCA Protein Assay Kit (Thermo Scientific) as instructed by manufacturer. Human DIPP was transformed, expressed and purified using same protocol described above.

### Nudix hydrolases activity tests

Nudix activity assays were performed at 37°C using 50 mM NaCl, 40 mM Hepes buffer (pH 7.4, unless stated otherwise), 0.25 micromoles of polyP_60_ or 5 nanomoles of 5-diphosphoinositol pentakisphosphate (IP_7_), 6 mM MgCl_2_ or other specified cation and about 0.5 µg/ml of recombinant protein for 1 h or indicated time. For enzymatic reactions at different pHs, we used MES buffer for pH 5.5–6.5, Hepes for pH 7.0–8.0 and Tris-base for pH 8.5. Enzymatic reactions were stopped by addition of 3 µl of 100 mM EDTA and kept on ice or frozen until further use. Products were resolved by polyacrylamide gel electrophoresis using 30 or 35% acrylamide/bis-acrylamide 19:1 (National Diagnostics) gels in Tris/Borate/EDTA (TBE) buffer as previously described [25]. Gels were then stained with toluidine blue for 1 h and de-stained on 20% methanol for several hours until background staining was removed. For kinetic measurements, the same activity test was performed at pH 8.0 using various quantities of indicated substrate for 10 min. Then P_i_ released from substrates was quantified by malachite green assay. First, we prepared reagent mix (0.045% malachite green with 4.2% ammonium molybdate in 4 M HCl at a 1:3 ratio, respectively) and let it sit for at least 10 min, and then filtered the solution with 0.2 µm syringe filter units (Millipore). We then added 100 µl of reagent mix to 100 µl of reaction in a clear 96-well plate, mixed well and immediately read absorbance at 660 nm. We quantified P_i_ through comparison with a standard curve made by serial dilution of KH_2_PO_4_. P_i_ concentration obtained was used for kinetic calculations and plotted in GraphPad Prism 6 software.

### Endogenous tagging

We generated cell lines with endogenous C-terminal tags using a one-step transfection method [26]. We amplified by PCR a cassette from pMOTag4H using primers that contained 80 nt homologous region of the 3′ end of CDS and 3′UTR of TbNH4 (Supplementary Table S1). The construct was verified by agarose gel electrophoresis, PCR purified using Minelute PCR purification kit (Qiagen), and transfected in *T. brucei* Lister 427 PCF cells. Transfection was performed as described before and cells were selected using hygromycin [27]. Preparation of culture lysates, and SDS/PAGE and western blot analyses using anti-HA antibody (Covance) were done to verify expression of tagged proteins [27]. We used microscopy to localize tagged proteins, sample preparation for immunofluorescence microscopy was done as described before [27].

### Construct for overexpression

The sequence of TbNH2 (Tb427.05.4350) was amplified by PCR using Q5^®^ high-fidelity polymerase (NED) and cloned in the vector pLEW100v5b1d-BSD using the Gibson Assembly^®^ Cloning kit (NEB) (Supplementary Table S1). Sequence was verified by Sanger sequencing and plasmid transfected in *T. brucei* procyclic 29-13 TetR/T7RNAP cell line. Cells were cultured for 2 days with tetracycline (1 µg/ml) for induction of overexpression. To verify overexpression, we extracted RNA, synthesized cDNA and performed by qRT-PCR as described previously [27]. Relative gene expression data were obtained by comparison with actin expression levels. We also used western blot analysis with specific antibody to validate increase in TbNH2 protein translation.

### Antibody production

We digested the recombinant protein construct of TbNH2 with thrombin (Sigma) to remove thioredoxin and the His-tag from the construct. We then applied products to a HIS-Select^®^ Cartridge column to remove tag from mixture, allowing us to collect pure TbNH2 in the flow-through. This protein was quantified using Pierce BCA Protein Assay Kit (Thermo Scientific) and used for antibody production. The antigen was injected to six female CD-1 mice (Charles River Laboratories) intraperitoneally. The primary inoculation contained 100 µg purified protein mixed in equal parts with Freund’s complete adjuvant (Sigma). Subsequent boosts, spaced in 2-week intervals, contained 50 µg purified protein mixed in equal parts with Freund’s incomplete adjuvant (Sigma). Final bleeds were collected via cardiac puncture.

### Fluorescence microscopy

*Trypanosoma brucei* PCF were centrifuged at 1000 × ***g*** for 10 min at 25°C; washed twice with PBS, pH 7.4; and fixed with 4% paraformaldehyde in PBS for 1 h at room temperature (RT). Afterward, cells were adhered to poly-L-lysine coated coverslips for 30 min; permeabilized with 0.1% Triton X-100 in PBS for 5 min, washed three times, and blocked with PBS containing 100 mM NH_4_Cl, 3% BSA, 1% fish gelatin, and 5% goat serum for 1 h. Cells were then incubated for 1 h, at RT, with primary antibodies: anti-HA tag monoclonal antibody (1:250), polyclonal mouse anti-NH2 antibody (1:1000), and polyclonal rabbit anti-PPDK antibody (1:30), as glycosomal marker. After washing three times with 3% BSA in PBS (pH 8.0), cells were incubated at RT in the dark with secondary antibodies: Alexa Fluor 488-conjugated goat anti-mouse (1:1000), or Alexa Fluor 546-conjugated goat anti-rabbit (1:1000). Then cells were counterstained with 5 µg/ml DAPI to label nuclei and kinetoplasts. Finally, all preparations were washed again three times with 3% BSA in PBS (pH 8.0) and mounted on glass slides with Fluoromount-G (Southern Biotechnology). Differential interference contrast (DIC) and fluorescence optical images were captured under non-saturating conditions and identical exposure times using an Olympus IX-71 inverted fluorescence microscope with a Photometrix Cool-SnapHQ charge-coupled device (CCD) camera driven by DeltaVision software (Applied Precision). Images were deconvolved for 15 cycles using Softwarx deconvolution software.

## Results

### Analysis of *T. brucei* Nudix hydrolase sequences

It has been reported [21] that five putative Nudix proteins are present in the *T. brucei* proteome: TbNH1 (Tb927.11.15640; MW: 44.4; isoelectric point [IP]: 5.49), TbNH2 (Tb927.5.4350; MW: 19.6; IP: 6.88), TbNH3 (Tb927.11.9810; MW: 27.5; IP: 4.83), TbNH4 (Tb927.6.2670; MW: 33.0; IP: 8.62) and TbNH5 (Tb927.10.4680; MW: 32.2; IP: 7.11). All of them have the Nudix box of 23 amino acids common to other NHs but have little identity with Ddp1 (16% for TbNH2 and 13% for TbNH4). TbNH1 (MERS) is a mitochondrial mRNA stability factor [20] and TbNH4 (TbNDcp2) has de-capping activity [21]. The activity of the other Nudix proteins has not been investigated. TbNH2 and TbNH3 has been localized to the glycosomes by proteomic studies [28].

### PolyP polyphosphatase activity of NHs from *T. brucei*

**Figure 1:**
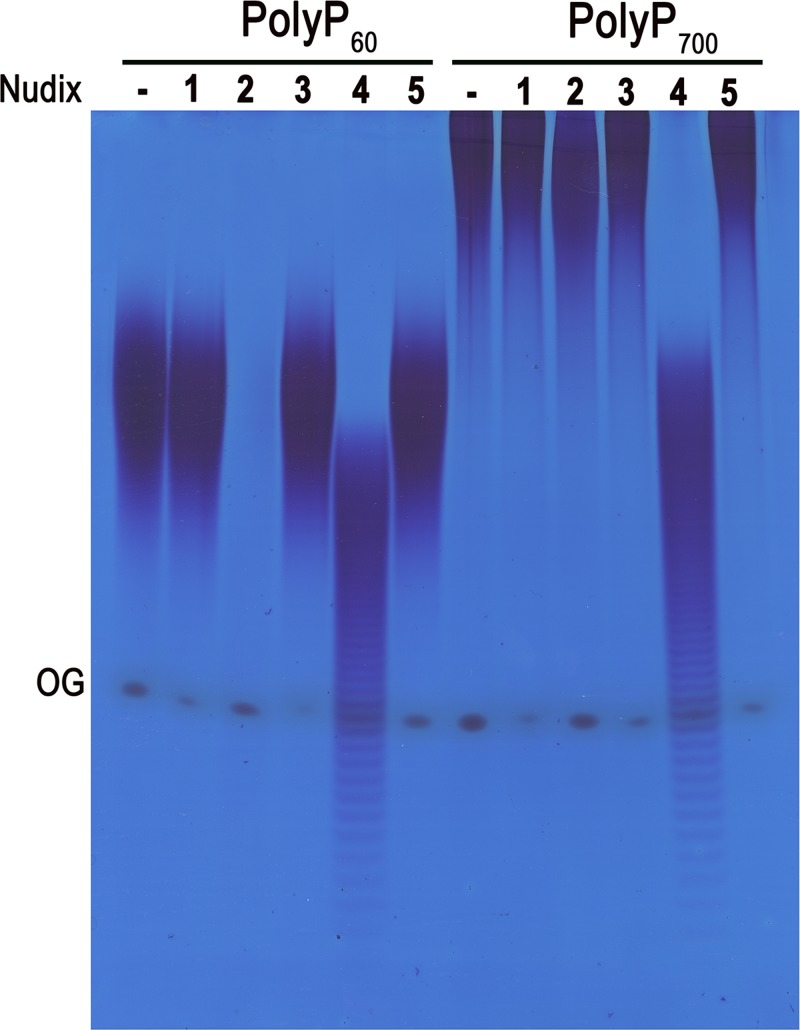
Screening of Nudix hydrolase activities identifies two polyP phosphatases in *T. brucei* The ability of the five NHs to degrade short (polyP_60_) and long chain polyP (polyP_700_) was tested at 37°C for 1 h in medium containing 40 mM Hepes buffer, pH 7.4, 50 mM NaCl, 6 mM MgCl_2_, 0.25 micromoles of polyP_60_ or polyP_700_ and 5 µg/ml of recombinant protein. Controls (-) and enzymatic products from NH 1, 2, 3, 4 and 5 were resolved in 30% polyacrylamide gels. TbNH2 has high activity with polyP_60_ and apparently no activity with polyP_700_. TbNH4 has activity with both polyP_60_ and polyP_700_, as evidenced by the production of shorter chain polyP. Orange G (OG) dye was used as loading indicator.

**Figure 2:**
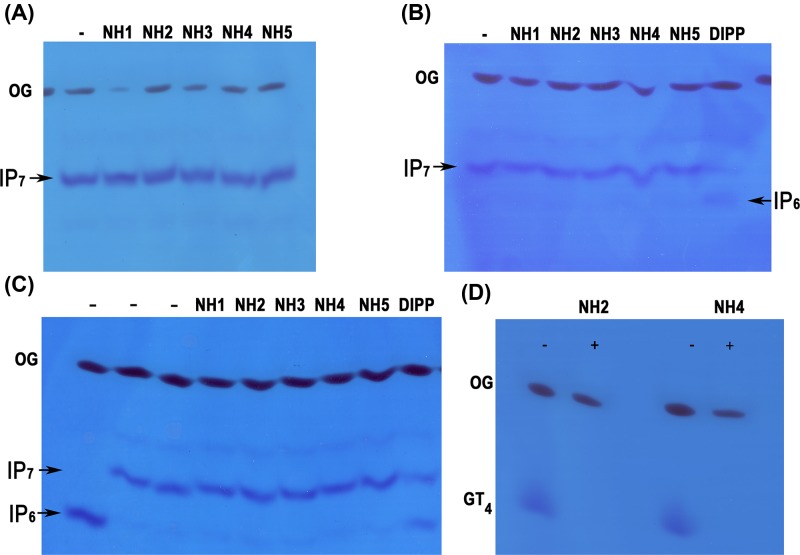
Lack of hydrolytic activity of TbNH2 and TbNH4 against 5-diphosphoinositol pentakisphosphate (5-IP_7_) at different pHs and activity against GP_4_ Phosphatase activity was assayed for 1 h at 37°C in medium containing 40 mM Hepes buffer, pH 7.4, 50 mM NaCl, 6 mM MgCl_2_, 5 nanomoles of 5-diphosphoinositol pentakisphosphate (5-IP_7_) and 5 µg/ml of recombinant protein, at pH 6 (**A**), 7 (**B**) or 8 (**C**). (**D**) Phosphatase activity against GT4. Assays were done as in (A–C), using 20 nanomoles GP4 instead of 5-IP_7_. The three first lanes in (C) do not have enzymes, and were loaded with IP_6_ (first lane) or IP7 (second and third lanes).

To test whether any of the five NHs from *T. brucei* has polyP polyphosphatase activity, we cloned and expressed them in bacteria with a polyhistidine tag, and purified the proteins using nickel columns, as described under Materials and Methods. We were able to obtain proteins of the expected size (Supplementary Figure S1), which were used on activity tests with polyPs of different sizes. Incubation of the enzymes with commercially available polyP_60_ showed a significant polyP hydrolyzing activity of TbNH2 and TbNH4 while neither TbNH1, TbNH3, nor TbNH5 was able to hydrolyze it. In contrast, only TbNH4 was able to hydrolyze polyP_700_ (Figure 1). None of the *T. brucei* NHs was able to hydrolyze 5-IP_7_ at either pH 6.0, 7.0 or 8.0 in contrast to human DIPP, used as positive control (Figure 2A–C). However, both TbNH2 and TbNH4 were able to degrade guanosine tetraphosphate (GP_4_) (Figure 2D).

### Characterization of TbNH2 activity

**Figure 3:**
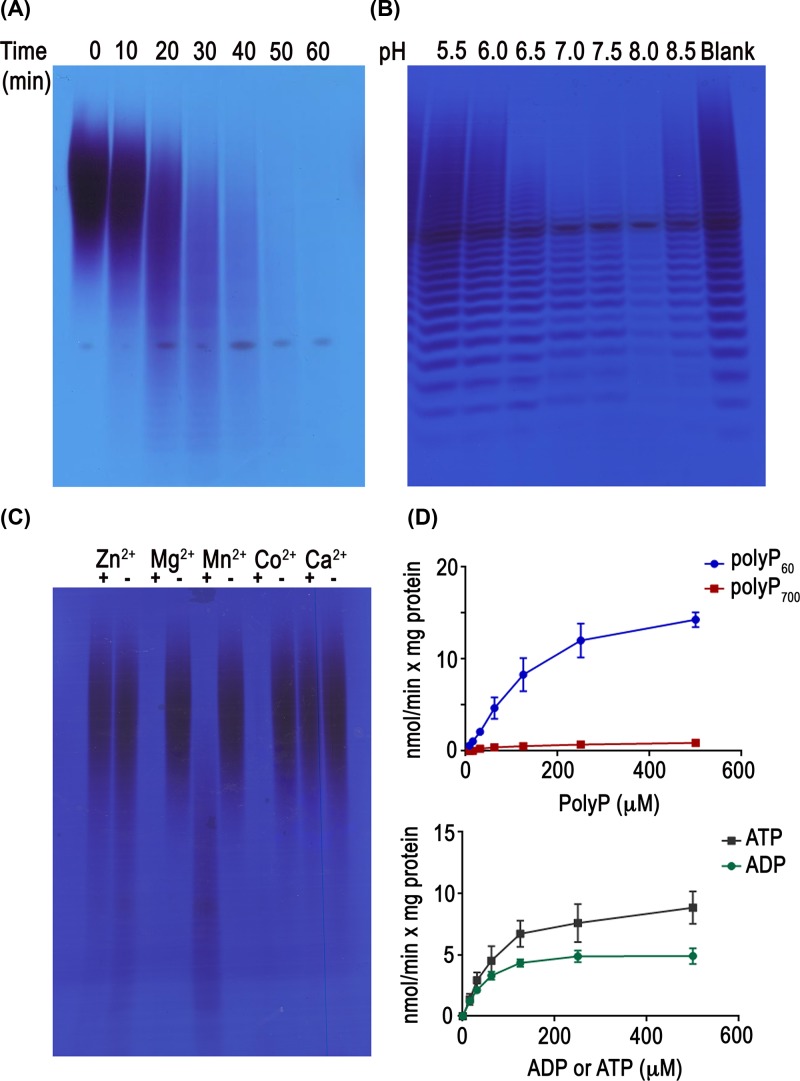
Characterization of TbNH2 activity (**A**) Hydrolysis of polyP_60_ by TbNH2 over 1 h was tested at 37°C in medium containing 40 mM Hepes buffer, pH 7.4, 50 mM NaCl, 6 mM MgCl_2_, 0.25 micromoles of polyP_60_ and 5 µg/ml of recombinant protein. (**B**) TbNH2 activity on polyP_60_ at various pHs. (**C**) TbNH2 activity on polyP_60_ in the presence of divalent cations (6 mM). (**D**) Phosphatase activity of TbNH2 with different concentration of polyP_60_, polyP_700_, ATP and ADP_._ Note that the activity on polyP_700_, is about ten times lower than the activity on polyP_60_.

We tested the activity of TbNH2 on polyP_60_ over the course of 1 h and resolved the products by PAGE (Figure 3A). The progressive shortening of the polyP polymer with production of P_i_ (see below) demonstrates an PPX activity. Yeast Ddp1 has been reported to have higher activity at slightly acidic pH, while human DIPPs have preference for higher pH [13]. TbNH2 has higher activity at basic pH 8.0 (Figure 3B). We also tested the activity of TbNH2 with different cofactors. TbNH2 is able to hydrolyze polyP with Mg^2+^ and Co^2+^ as cofactors, but has lower activity with Mn^2+^ (Figure 3C). We used the malachite green assay to compare P_i_ release from polyP, ATP and ADP. TbNH2 has a higher affinity for polyP_700_ than for polyP_60_ (*K*_m_ = 125.6 ± 45 µM versus 200.5 ± 36 µM with *V*_max_ = 1.1 ± 0.1 nmol min^−1^ mg protein^−1^ versus 20.6 ± 1.7 nmol min^−1^mg protein^−1^) (Figure 3D, top and Table 1). This difference in affinity based on polyP size suggests that the enzyme works by removing the terminal phosphate from polyP chains exerting an PPX activity. Interestingly, using same method we detected TbNH2 also has activity to release the γ and β phosphates from ATP and ADP (Figure 3D, bottom).

**Table 1:** Kinetic parameters of PPX activity of TbNH2 and TbNH4

Enzyme	Substrate	*V*_max_ (nmol min^−1^ mg^−1^)	*K*_m_ (µM)	*k*_cat_*/K*_m_ (s^−1^M^−1^)
TbNH2	PolyP_60_	20.6 ± 1.7	200.5 ± 36	2.2 × 10^5^
	PolyP_700_	1.1 ± 0.1	125.6 ± 45	0.2 × 10^5^
	ADP	5.6 ± 0.2	46.5 ± 6.3	2.7 × 10^5^
	ATP	10.2 ± 0.7	78.1 ± 16.3	2.9 × 10^5^
TbNH4	PolyP_60_	9.1 ± 0.4	82.2 ± 13	3.4 × 10^5^
	PolyP_700_	5.6 ± 0.2	149.4 ± 21	1.1 × 10^5^

### Characterization of TbNH4 activity

**Figure 4:**
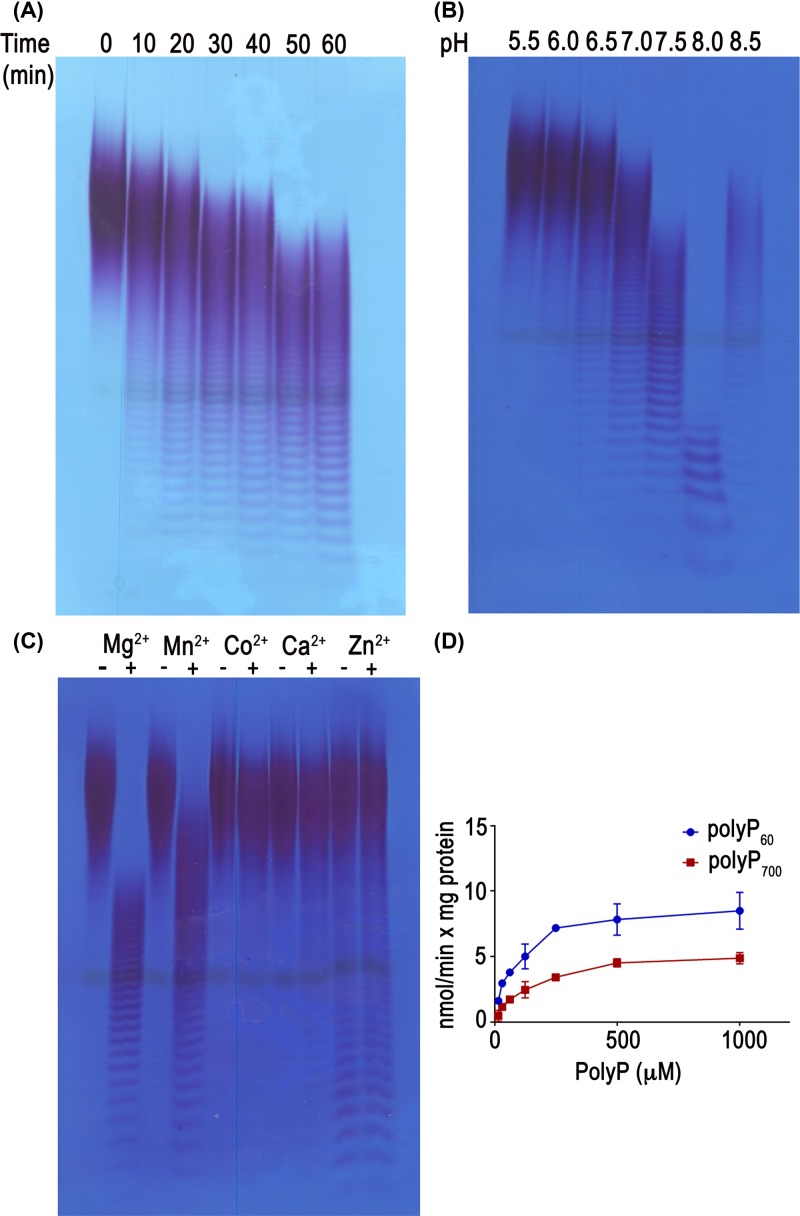
Characterization of TbNH4 activity (**A**) Hydrolysis of polyP_60_ by TbNH4 over 1 h was tested at 37°C in medium containing 40 mM Hepes buffer, pH 7.4, 50 mM NaCl, 6 mM MgCl_2_, 0.25 micromoles of polyP_60_ and 5 µg/ml of recombinant protein. (**B**) TbNH4 activity on polyP_60_ at various pHs. (**C**) TbNH4 activity on polyP_60_ in the presence of divalent cations. (**D**) Phosphatase activity of TbNH4 with different concentration of polyP_60_ and polyP_700_.

We also tested the activity of TbNH4 on polyP_60_ over the course of 1 h (Figure 4A). TbNH4 endopolyphosphatase activity was shown by the increase in the staining intensity of small polyP oligomers that were produced after incubation of polyP_60_ with the enzyme. TbNH4 has a slightly lower activity on polyP_60_ than TbNH2. Activity tests at various pHs showed TbNH4 has optimum activity at pH 8.0 (Figure 4B). Mg^2+^ or Mn^2+^ were the preferred cofactors while no activity was detected with Co^2+^ (Figure 4C). The malachite green assay for detection of P_i_ release showed that the enzyme also has exopolyposphatase activity and a higher affinity for polyP_60_ than for polyP_700_ (Figure 4D and Table 1) (*K*_m_ = 82.1 ± 13 µM versus 149.4 ± 21 µM and *V*_max_ = 9.1 ± 0.4 nmol min^−1^mg protein^−1^ versus = 5.6 ± 0.2 nmol min^−1^mg protein^−1^). TbNH4 does not have phosphatase activity against ATP or ADP.

### Localization studies

**Figure 5:**
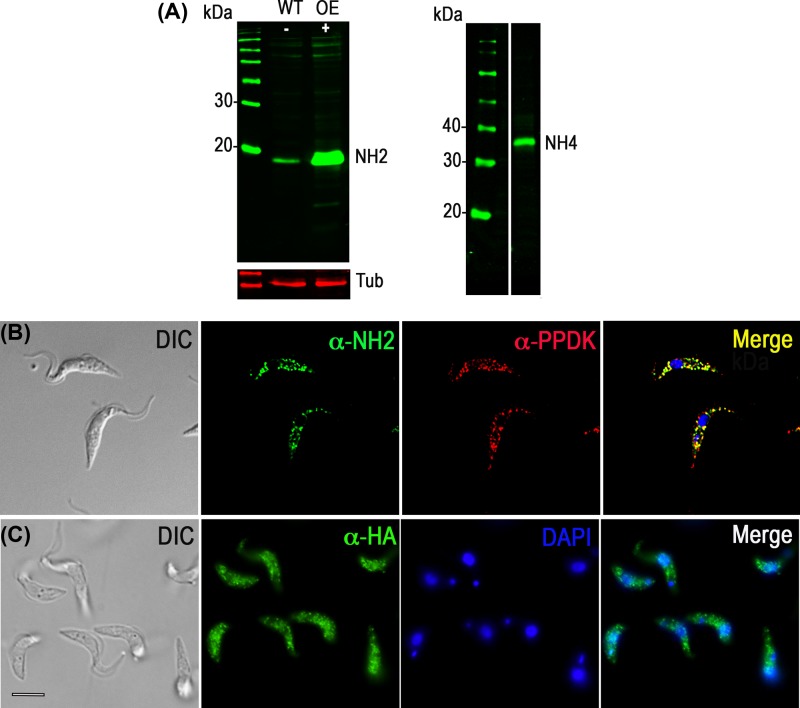
Subcellular localization of TbH2 and TbNH4 (**A**) Left panel, western blot analysis of wild type or TbNH2 overexpressing cells using polyclonal anti-TbNH2 antibody showing a band of about 19 kDa. Antibodies against α-Tubulin (Tub) were used as loading control. Right panel, western blot analysis of endogenously tagged parasites using monoclonal anti-HA antibodies showing TbNH4 (37 kDa). Molecular weights are shown on the left. (**B**) TbNH2 co-localizes with PPDK in glycosomes of PCF. TbNH2 was detected with polyclonal anti-TbNH2 antibody in cells overexpressing the protein (green), and co-localized with antibodies against PPDK (red). The merge shows co-localization in yellow. (**C**) TbNH4 localizes in the cytosol and nucleus of PCF. TbNH4 was detected with monoclonal anti-HA antibodies in trypanosomes expressing TbNH4-HA (green). A punctate appearance could be the result of deconvolution of the images. Bar (for **B** and **C**) = 5 µm.

Trypanosomes accumulate large amounts of polyP in acidocalcisomes [4]. PolyP has also been found in glycosomes and nucleoli of *T. brucei* [22]. In order to determine the localization of TbNH4, we tagged the C-terminus of the gene with an HA tag using homologous recombination with the endogenous gene locus in procyclic trypomastigotes (PCF). Western blot analysis using anti-HA antibodies confirmed the expression of the protein of the expected size (37 kDa, Figure 5A right panel). Because the tag could interfere with the glycosomal localization signal of TbNH2, we prepared a specific polyclonal antibody and overexpressed the protein. Western blot analysis using this antibody labeled a protein of the expected size (19 kDa, Figure 5A, left panel). Immunofluorescence microscopy showed that overexpressed TbNH2 localized to the glycosomes, as demonstrated by co-localization with the glycosomal marker PPDK (Figure 5B). Immunofluorescence microscopy shows that tagged TbNH4 localizes to the cytosol and nuclei of the cells (Figure 5C). No fluorescence was observed in control parasites incubated only in the presence of secondary antibodies (not shown).

## Discussion

The most important findings of this work are the identification of two NHs of *T. brucei* as polyP phosphatases, and evidence of their localization in subcellular organelles where polyP is or may be present. None of the other three NHs of the parasites has this activity, and none of the NHs can hydrolyze 5-IP_7_.

TbNH4 has polyP endopolyphosphatase activity, the ability to attack internal phosphoanhydride bonds hydrolyzing polyP molecules into smaller oligophosphates, and is the first such activity identified in trypanosomatids. TbNH4 has also PPX activity, the ability to remove P_i_ from the end of polyP chains, as the yeast PPN1 endopolyphosphatase previously described [29]. TbNH4 PPX has higher affinity for polyP_60_ than for polyP_700_. However, this could be attributed to the detection method. Although the enzyme can hydrolyze phosphoanhydride bonds at any position, only P_i_ is detected by the malachite green assay used and shorter polyP chains have more available ends to release P_i_ than polyP of longer chains. TbNH4 localizes to the cytosol and nucleus, as occurs with Ddp1 [16]. PolyP has been shown to be cytotoxic when in the yeast cytosol [30] and this enzyme, together with TbPPX [5], could help in controlling its cytosolic concentration. The cytosolic localization could also be important for its de-capping activity [21] and the nuclear localization could be relevant to regulate nucleolar polyP levels in procyclic forms [22].

TbNH2 has polyP PPX activity and has a preference for short chain polyP. PolyP is a linear polymer of phosphate, so there is no chemical difference among polyPs of different chain lengths except for the number of ends available for the PPX to hydrolyze. The convention is to quantify the amount of polyP by molarity of phosphate units. Therefore, polyP_60_ and polyP_700_ at the same molarity have the same amount of phosphate units, but not the same number of molecules. The activity of a PPX should be the same only if there is the same number of molecules in solution. Actually, the number of polyP molecules available on polyP_60_ is 11.7 lower than for polyP_700_, and our assays show about 18 times reduction in *V*_max_ between polyP_60_ and polyP_700_. This result supports the activity of TbNH2 as a PPX.

TbNH2 was reported in two glycosome proteomes [28,31], has a peroxisomal targeting signal 2 (PTS2) and, as other peroxisomal NHs, has been proposed to have a role in destroying nucleotides damaged by reactive oxygen species [15]. Interestingly, expression of a yeast PPX in the glycosomes of *T. brucei* makes them more susceptible to oxidative stress [22] and polyP has been shown to have a role in protection against oxidative stress [32,33]. It would be interesting to test whether overexpression of an endogenous gene, which could be susceptible to regulatory mechanisms not present when overexpressing an exogenous gene, have the same effect.

Alkaline pH and divalent ions have been found before to be important for Nudix hydrolase activity [18]. Both TbNH2 and TbNH4 have similar preferences for alkaline pH and divalent cofactors (Mg^2+^ and Co^2+^ for TbNH2 and Mg^2+^ and Mn^2+^ for TbNH4).

In conclusion, we have identified that two of the five NHs present in *T. brucei* have polyP endo- and exopolyphosphatase activities but, in contrast to the yeast and mammalian NHs, are unable to hydrolyze 5-IP_7_. TbNH2 is a polyP PPX, and localizes in the glycosome. TbNH4 is a polyP endo- and exopolyphosphatase, and localizes in the cytosol and nucleus. Both enzymes could have a role in maintaining and regulating polyP levels in their respective localizations.

## Supporting information

**Supplementary Figure 1:** 

**Supplementary Table S1:** Primers used in this study.

## Abbreviations

5-IP_7_: 5-Diphosphoinositol pentakisphosphate
Ddp1: diphosphoinositol polyphosphate phosphohydrolase
DIPP: diphosphoinositol polyphosphate hydrolase
GP_4_: guanosine tetraphosphate
IP: isoelectric point
NH: nudix hydrolase
P_i_: orthophosphate
polyP: inorganic polyphosphate
PP_i_: inorganic pyrophosphate
PPDK: phosphate pyruvate dikinase
TbNH: *T. brucei* Nudix hydrolases
RT: room temperature
VTC: vacuolar transporter chaperone
