# Supplementary material for: NUDIX hydrolases with inorganic polyphosphate exo- and endopolyphosphatase activities in the glycosome, cytosol and nucleus of Trypanosoma brucei
Source: Biosci Rep. 2019 May 17;39(5):BSR20190894. doi: 10.1042/BSR20190894 (PMC6522730; doi:10.1042/BSR20190894)
Supplement: Supplementary file 1 [file bsr20190894_Supp1.pdf]

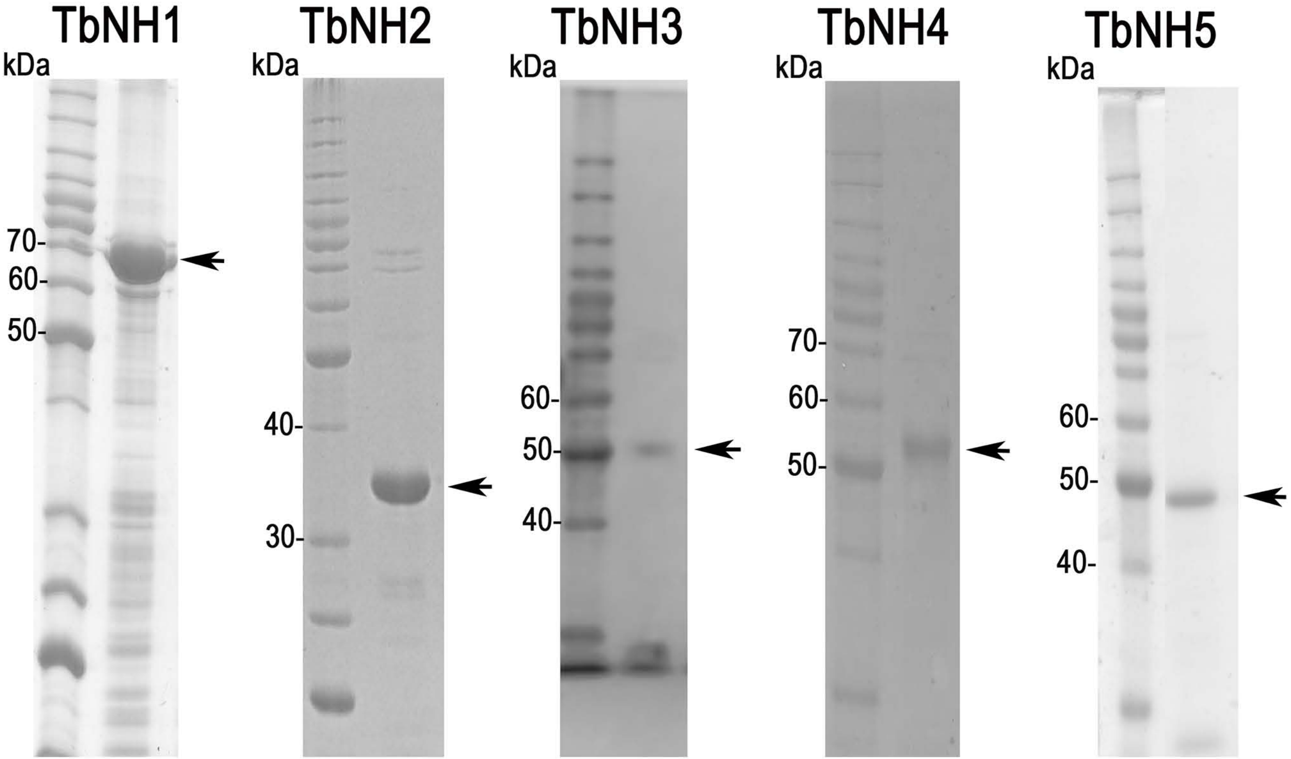
TbNH1
TbNH2
TbNH3
TbNH4
TbNH5
kDa
70-
60-
50-
40-
30-
60-
50-
40-
70-
60-
50-
60-
50-
40-

**Table S1.** Primers used in this study.

| Primer | Use |
|---|---|
| GACGACGACAAGATGCGCAAGCAATTATTTTTC | (fwd); cloning of TbNH1 in pET32 |
| GAGGAGAAGCCCGGTTCACGATGCATCTTCCC | (rev); cloning of TbNH1 in pET32 |
| GACGACGACAAGATGTACCGAAAAAATGTATGTGTGGTA | (fwd); cloning of TbNH2 in pET32 |
| GAGGAGAAGCCCGGTTTATATGGAAGACCTTGTTTTCAAAA ATGC | (rev); cloning of TbNH2 in pET32 |
| GACGACGACAAGATGAGGGACCGCTATATCAG | (fwd); cloning of TbNH3 in pET32 |
| GAGGAGAAGCCCGGTTCAGAGAGTTGCTATTTTCAGAC | (rev); cloning of TbNH3 in pET32 |
| GACGACGACAAGATGCCAAACGAAACCGCGG | (fwd); cloning of TbNH4 in pET32 |
| GAGGAGAAGCCCGGTTCACTCGCAGGAGGTGGG | (rev); cloning of TbNH4 in pET32 |
| GACGACGACAAGATGGCTATGGGAAGAGTTT | (fwd); cloning of TbNH5 in pET32 |
| GAGGAGAAGCCCGGTCTAACGCTTTCCAAGTGGG | (rev); cloning of TbNH5 in pET32 |
| AATCAATTGTTACATGAGGAGGGAGGTTCCCCTCAGATGA ATTTACTTGGAAACCCCACCTCCTGCGAGCGAGACGGGTGG GGGTTTTCGGGTACCGGGCCCCCCCTCGAG | (fwd); C-terminal tagging of TbNH4; template pMOTag4H |
| CGAGGAGCCAAATGCAAAGAGGATATTAAACATTATTTTT ATGCGATGTTACAATGCCACTGTGAGAGCTATACGAGGTGT CGTTTTTCTTGGCGGCCGCTCTAGAACTAGTGGAT | (rev); C-terminal tagging of TbNH4; template pMOTag4H |
| TAAAATTCACAAGCTTATGTACCGAAAAAATGTATGTGTGG TAATA | (fwd); cloning of TbNH2 in pLEW100v5b1d-BSD |
| TAAATGGGCAGGATCCTTATATGGAAGACCTTGTTTTCAAA A | (rev); cloning of TbNH2 in pLEW100v5b1d-BSD |
